# A Combination of Genetic Algorithm and Particle Swarm Optimization for Vehicle Routing Problem with Time Windows

**DOI:** 10.3390/s150921033

**Published:** 2015-08-27

**Authors:** Sheng-Hua Xu, Ji-Ping Liu, Fu-Hao Zhang, Liang Wang, Li-Jian Sun

**Affiliations:** Research Center of Government GIS, Chinese Academy of Surveying and Mapping, 28 Lianhuachi West Road, Haidian District, Beijing 100830, China; E-Mails: liujip@casm.ac.cn (J.-P.L.); zhangfh@casm.ac.cn (F.-H.Z.); wangl@casm.ac.cn (L.W.); sunlj@casm.ac.cn (L.-J.S.)

**Keywords:** vehicle routing problem, VRPTW, particle swarm optimization, genetic

## Abstract

A combination of genetic algorithm and particle swarm optimization (PSO) for vehicle routing problems with time windows (VRPTW) is proposed in this paper. The improvements of the proposed algorithm include: using the particle real number encoding method to decode the route to alleviate the computation burden, applying a linear decreasing function based on the number of the iterations to provide balance between global and local exploration abilities, and integrating with the crossover operator of genetic algorithm to avoid the premature convergence and the local minimum. The experimental results show that the proposed algorithm is not only more efficient and competitive with other published results but can also obtain more optimal solutions for solving the VRPTW issue. One new well-known solution for this benchmark problem is also outlined in the following.

## 1. Introduction

The vehicle routing problem (VRP) is a combinatorial optimization and integer programming problem seeking to service a number of customers with a fleet of vehicles. Proposed by Dantzig and Ramser in 1959, VRP is important to the fields of transportation, scheduling, distribution and logistics [1,2]. The problem involves many real-world considerations, such as time-window constraints, driver waiting costs, backhauls, layovers, *etc.* The vehicle routing problem with time windows (VRPTW) has been extensively studied by many researchers from the fields of operational research, applied mathematics, network analysis, graph theory, computer applications, traffic transportation, *etc.* Firstly, VRPTW is still one of the most difficult problems in combinatorial optimization, consequently presenting a great challenge. Secondly, in a more practical aspect, study of this problem could provide a real opportunity to reduce the costs in the area of logistics [3]. 

The VRPTW is a generalization of the VRP where the service for a customer starts within a given time interval, and it has been the subject of intensive research efforts for both heuristic and exact algorithms. The actual solutions of VRP can be generally classified into two main categories: the exact algorithms and the heuristic algorithms. The main approaches for solving VRPs are shown in Table 1. 

**Table 1:** Main approaches for solving VRPs.

Algorithms			Remarks
The exact algorithms	Branch and bound method [4,5]		The Efficiency depends on the depth of the branch and bound tree.
	Set segmentation method [6,7]		Hard to determine the minimum cost for each solutions.
	Dynamic programming method [8,9]		Effective to limited-size problems, hard to consider the concrete demands such as time windows.
	Integer programming algorithm [10,11]		High precision, time consuming, complex.
The heuristic algorithms	The traditional heuristic algorithms	Savings algorithm [12,13]	Computes rapidly, hard to get the optimal solution.
		Sweep algorithm [14,15]	Suitable to the same number of customers for each route with few routes.
		Two-phase algorithm [16,17]	Hard to get the optimal solution.
	The meta-heuristic algorithms	Tabu search algorithm [18,19,20]	Has the good ability of local search, but is time consuming, and depends on the initial solution.
		Genetic algorithm [13,21]	Has the good ability of global search, computes rapidly, hard to obtain the global optimal solution.
		Iterated local search [22,23]	Has the strength of fast convergence rate and low computational complexity.
		Simulated annealing algorithm [24,25]	Slow convergence rates, carefully chosen tunable parameters.
		Variable neighborhood Search [26,27]	Is suitable for large and complex optimization problems with constraints.
		Ant colony algorithm [28,29,30]	Has good positive feedback mechanism, but is time consuming and prone to stagnation.
		Neural network algorithm [31,32]	Computes rapidly, has slow convergence and can easily be trapped in a local optimum
		Artificial bee colony algorithm [30,33]	Achieves a fast convergence speed, is associated with the piecewise linear cost approximation.
		Particle swarm optimization [34,35,36]	Is robust and has fast searching speed, brings easily premature convergence.
		Hybrid algorithm [2,8,12,20,28,37,38]	Is simple with fast optimizing speed and less calculation.

The exact algorithms can obtain the exact solution, but the computational effort required to solve this problem increases exponentially with the problem size. The traditional heuristic algorithms often only get the approximate solution close to the optimal solution, and are limited to the smaller problems. When the size of the problems increases, the solution precision of the traditional heuristic algorithms is often poor. The traditional heuristic algorithms adapt to local optimization and combined with the meta-heuristic algorithms to improve the solutions [39]. For large, complex problems, only the meta-heuristic algorithms can be used due to their strong performance of global search [18,40,41].

The VRPTW is a non-deterministic polynomial-time hard (NP-hard) problem. Due to the complexity of the VRPTW, it is not easy to obtain an exact solution for a large problem in real time. For such problems, optimal solutions are found quickly and are sufficiently accurate. Usually this task is accomplished by using various meta-heuristic algorithms, which rely on some insights into the nature of the problem. Particle swarm optimization (PSO) is not only superior in terms of high accuracy speed calculation, as well as its simple program, but it is also robust. Nie and Yue integrated the concept of evolving individuals originally modeled by GA with the concept of self-improvement of PSO [42]. Hao *et al.* proposed a modified particle swarm optimization which took the crossover between each particle’s individual best position [43]. In [44], dynamic parameterized mutation and crossover operators were combined with a PSO implementation individually and in combination to test the effectiveness of these additions. In [45], the proposed method used the concept of particles’ best solution and social best solution in the PSO algorithm, followed by combining it with crossover and mutation of GA. Considering the particle real encoding, linear decreasing inertia weight function and crossover operator of genetic algorithms, a combination of genetic algorithm and PSO for VRPTW is proposed that can improve the performance when computing speed to obtain the optimal solutions. 

## 2. Vehicle Routing Problem with Time Windows (VRPTW)

In the VRPTW, a fleet of K identical vehicles supplies goods to n customers. All the vehicles have the same capacity Q. For each customer i, i=1,2,⋯,n, the demand of goods $d_{i}$, the arrival time $t_{i}$, the service time $s_{i}$, the waiting time $w_{i}$ and the time window $\left[b_{i},e_{i}\right]$ to meet the demand in customer i are all decision variables. The component $s_{i}$ represents the loading or unloading service time for customer i, whereas $b_{i}$ describes the earliest time available for starting the service. If any vehicle arrives at customer i before $b_{i}$, it must wait. The vehicle must start the customer service before $e_{i}$. This type of time window constraint is well known as a hard time window. Each of the vehicle routes starts and finishes at the central depot. Correspondingly, each customer must be visited once. The locations of the depot and all customers, the minimal distance $c_{ij}$ and the travel time $t_{ij}$ between all locations are given.

From the perspective of the graph theory, the VRPTW can be stated as follows: Let G(V,A) be an undirected graph with a node set $V=(v_{0},v_{1},\cdots ,v_{n})$ and an arc set $A=\{(c_{i},c_{j}):i\neq j,c_{i},c_{j}\in C\}$. In this graph model, $c_{0}$ is the depot, $c_{i}(i=1,2,\cdots ,n)$ is a customer. To each arc $\left(c_{i},c_{j}\right)$ is associated a value $t_{ij}$ representing the travel time from $c_{i}$ to $c_{j}$. A route is defined as starting from the depot, going through a number of customers and ending at the depot; each customer $c_{i}(i=1,2,\cdots ,n)$ must be visited exactly once. There are K vehicles, $V_{e}=\{0,1,\cdots ,K-1\}$. The number of routes cannot exceed K. Each vehicle serves a subset of customers on the route. The vehicles have the same capacity Q. The total sum of demands of customers served by a vehicle on a route cannot exceed Q. The additional constraints are that the service beginning time at each node $c_{i}(i=0,1,\cdots ,n)$ must be greater than or equal to $b_{i}$, the beginning of the time window, and the arrival time at each node $c_{i}$ must be lower than or equal to $e_{i}$, *i.e.*, the end of the time window. In case the arrival time is less than $b_{i}$, the vehicle has to wait until the beginning of the time window before starting servicing the customer. The goal is to find a set of routes which can guarantee each customer to be served by one vehicle within a given time interval and then satisfy the vehicle capacity constraints. Furthermore, the size of the set should be less than the number of vehicles needed and the total travel distance should be minimized. Moreover, the mathematical formulation of the VRPTW is presented as follows [4,46]:

(1) $$Minz=\sum_{i=0}^{n}\sum_{j=0}^{n}\sum_{k=0}^{K-1}c_{ij}x_{ijk}$$

Subject to:

(2) $$\sum_{k=0}^{K-1}\sum_{j=1}^{n}x_{ijk}\leq K(i=0)$$

(3) $$\sum_{k=0}^{K-1}\sum_{j=0}^{n}x_{ijk}=1(i=1,2,\cdots n;i\neq j)$$

(4) $$\sum_{i=0}^{n}d_{i}\sum_{j=0}^{n}x_{ijk}\leq Q(i\neq j,\forall k\in [0,K-1])$$

(5) $$t_{0}=0$$

(6) $$t_{i}+t_{ij}+s_{i}-M(1-x_{ijk})\leq t_{j}(i,j\in [1,n];i\neq j;k\in [0,K-1])$$

(7) $$b_{i}\leq t_{i}\leq e_{i}$$

(8) $$\sum_{i=0}^{n}x_{ihk}-\sum_{j=0}^{n}x_{hjk}=0(j\in V,k\in V_{e})$$

where $x_{ijk}$ is 1 if vehicle k travels from customer i to customer j, and 0 otherwise. $t_{i}$ denotes the time a vehicle starts services at customer i; M is an arbitrary large constant. Objective function states that the total cost is to be minimized. Constraint (2) specifies that there are no more than K routes going out of the depot. Constraint (3) ensures that one vehicle goes into and out of a customer exactly. Equation (4) is the capacity constraint. The time window is assured in Equation (5), Equation (6) and Equation (7). Equation (8) is the flow conservation constraints that describe the vehicle path.

## 3. Particle Swarm Optimization (PSO)

PSO is a population-based stochastic optimization technique developed by Eberhart and Kennedy in 1995, inspired by social behavior of bird flocking or fish schooling [34] and was first intended for simulating these organisms’ social behavior. It is best to imagine a swarm of birds that are searching for food. When one of them finds the food, some of them will follow the first bird, while others will find other food. Initially, the birds do not know where the food is, but they know at each time how far the food is. Each bird will follow the one that is nearest to the food. Throughout the course of preying, a bird will use its own experiences and collective information to search for food. 

In particle swarm optimization, the particles are moved around in the search space according to a few simple formulae. The position of a particle represents a candidate solution to the optimization problem at hand. Each particle searches for better positions in the search space by changing its velocity according to rules originally inspired by behavioral models of flocking. The movements of the particles are guided by their own best-known position in the search space as well as the entire swarm’s best-known position. When improved positions are discovered, these will then come to guide the movements of the swarm. The process is repeated and by doing so it is hoped, but not guaranteed, that a satisfactory solution will eventually be discovered. Compared with other intelligence optimization algorithms such as ant colony optimization, genetic algorithm, simulated annealing algorithm, neural network algorithm and artificial immune algorithm, PSO retains the global search strategy based on the swarm and has no individuals as in crossover and mutation. In PSO, through adjusting the velocities and positions of the particles which fly through the problem space by following the current optimum particles, the optimal solution can be obtained. Due to its simplicity, strong robustness, and fast optimization speed, PSO is suitable for very large and complex optimization problems with constraints. At first, PSO was applied to solve continuous optimization problems; however, several applications were proposed during these years in the area of combinatorial optimization problems including shop scheduling [47,48], project scheduling [49], travelling sales force [50], partitional clustering [51], and vehicle routing [34,52]. 

PSO is one of the evolution algorithms with the characteristics of evolutionary computing and swarm intelligence. Similar to other evolutionary algorithms, PSO searches for optima by evaluating individual fitness based on cooperation and competition between individuals. In PSO, each individual is considered as a particle without weight and volume in n-dimensional search space and flies through the space with a certain speed. The speed is adjusted dynamically by the individual’s experience and the entire swarm’s experience. 

PSO is initialized with a population of random solutions and searches for the optimal solution by updating the particle’s position. Each particle is the feasible solution and is designated a fitness value by the objective function. Each particle keeps the track of its coordinates in the problem space which are associated with the best solution (fitness) it has achieved so far. The fitness value is called $p_{best}$. When a particle takes all the population as its topological neighbors, the best position is a global best and is called $g_{best}$. Through $p_{best}$ and $g_{best}$, particles update themselves to produce the next generation of swarms.

The selection of fitness function depends on the research goals. The fitness function to evaluate the individuals is always related to the objective function. For a VRPTW, the total cost can be viewed as the fitness value. The inverse of the total cost is used to represent the fitness of the individuals, and then the fitness function is defined as follows:

(9) $$f_{fitness}=\frac{1}{z}$$

In a PSO algorithm the particles represent potential solutions to the problem, and the swarm consists of P particles. Each particle p can be represented through n-dimensional vectors: the first one is defined by $X_{p}^{t}=(x_{p1}^{t},x_{p2}^{t},\cdots ,x_{pn}^{t})$ with p=(1,2,⋯,P) that indicates the position of the particle p in the searching space at the iteration t. The second one is $V_{p}^{t}=(v_{p1}^{t},v_{p2}^{t},\cdots ,v_{pn}^{t})$ that represents the velocity with which the particle p moves. The third one is $P_{best_{p}}^{t}=(p_{best_{p1}}^{t},p_{best_{p2}}^{t},\cdots ,p_{best_{pn}}^{t})$ that denotes the best position of the *p*th particle and the last one is $G_{best_{t}}^{t}=(g_{best_{p1}}^{t},g_{best_{p2}}^{t},\cdots ,g_{best_{pn}}^{t})$ that represents the global best position in the swarm until *t*th iteration. The swarm is updated by the following equations:

(10) $$\{\begin{array}{c} {v^{t+1}}_{pn}={v^{t}}_{pn}+k_{1}\times rand1()\times ({p^{t}}_{best_{pn}}-{x^{t}}_{pn})+k_{2}\times rand2()\times ({g^{t}}_{best_{pn}}-{x^{t}}_{pn})\\ {x^{t+1}}_{pn}={x^{t}}_{pn}+{v^{t+1}}_{pn} \end{array}$$

where $k_{1}$ and $k_{2}$ are acceleration coefficients, which are respectively called cognitive and social parameter; rand1() and rand2() are two random numbers uniformly distributed in [0,1]. Acceleration coefficients $k_{1}$ and $k_{2}$ are positive constants to control how far a particle will move in a single iteration. Low values allow particles to roam far from target regions before being tugged back, while high values result in abrupt movement towards, or past, target regions. Typically, these are both set to a value of 2.0, although assigning different values to $k_{1}$ and $k_{2}$ sometimes leads to an improved performance.

A constant, $v_{\max}$, is used to arbitrarily limit the velocities of the particles ${v^{t}}_{pn}$ and improve the resolution of the search. When $v_{\max}$ is large (≥5), the velocity of the particle is large, too; it is conducive to a global search, though it may fly through the optimal solution [53,54,55]. When $v_{\max}$ is small (≤0.3), the velocity of the particle is also small; it leads to a fine search in a specific region, but it is easy to fall to local optimum [53,54,55]. In a word, the search efficiency depends on $v_{\max}$.

Each particle moves in the search space with a velocity according to its own previous best solution and its group’s previous best solution. In Equation (10), the velocity ${v^{t+1}}_{pn}$ consists of three parts—momentum, cognitive and social parts—respectively each term of the right side of Equation (10). The momentum part denotes the previous velocity of the particle, which improves the ability of the global search. The cognitive part denotes the process of learning from an individual’s experience. The social part denotes the process of learning from others’ experience, which represents the information sharing and social cooperation between particles. The balance among these parts determines the performance of a PSO algorithm. Without the momentum part, the particle then moves in each step without knowledge of the past velocity. Without the cognitive part, the convergence speed is fast, but can easily fall into a local optimum for a large problem size. Without the social part, it is hard to get the optimal solution due to the lack of the communications among individuals. 

## 4. The Proposed Algorithm

### 4.1. Particle Encoding

Encoding is a bridge connecting a problem with an algorithm. Encoding method and initial solution have a great impact on the VRP problem. It is the key step to finding the appropriate encoding method for the particles and the corresponding solutions. In brief, the encoding methods of PSO include real encoding, binary-encoding and integer encoding. Integer encoding is easy to decode and convenient for the fitness function calculation, but it requires a lot of computing resources and tends toward premature convergence. In addition, binary encoding is hard to decode. Therefore, real encoding is adopted in the proposed method.

### 4.2. Inertia Weight Function

In order to better control the searching and exploring abilities and improve the convergence of particle swarm algorithm, the inertia weight ω is introduced into the velocity function. Therefore, ${v^{t+1}}_{pn}$ is changed into the following form:

(11) $${v^{t+1}}_{pn}=\omega {v^{t}}_{pn}+k_{1}\times rand1()\times ({p^{t}}_{best_{pn}}-{x^{t}}_{pn})+k_{2}\times rand2()\times ({g^{t}}_{best_{pn}}-{x^{t}}_{pn})$$

The inertia weight ω is employed to control the impact of the previous history of velocities on the current one. Accordingly, the parameter ω regulates the trade-off between the global and the local exploration abilities. If ω is high, particles can hardly change their direction and turn around, which consequently implies a larger area of exploration as well as a reluctance against convergence towards optimum. On the other hand, If ω is small, only little momentum is presented from the previous time-step, thereby leading to quick changes of direction. A suitable value for the inertia weight usually contributes the balance between global and local exploration abilities and consequently results in a reduction of the number of iterations required to obtain the optimum solution.

Considering whether the inertia weight ω is changed or not, the calculation methods of the inertia weight ω include three categories: fixed-weight method, time-varying weight method and adaptive inertia weight method. The fixed-weight method selects a constant value as the inertia weight ω and is kept unchanged. The fixed-weight method was originally introduced by Shi and Eberhart in [56]. The time-weight method selects an iterative relationship as the inertia weight ω according to a selected range, which is defined as a function of time or iteration number. In [57,58,59,60], time-varying inertia weight strategies were introduced and shown to be effective in improving the fine-tuning characteristic of the PSO. In [61,62,63], the adaptive inertia weight methods used a feedback parameter to monitor the state of the algorithm and adjusted the value of the inertia weight. 

In order to better balance the global search ability and local search capabilities, kinds of inertia weight are often used. At first, a higher ω is selected to expand the search space and converge to a region. Then a smaller ω is selected to explore the local region to obtain high accuracy solutions. In this paper, the definition of inertia weight is a linear decreasing function of the number of iterations. ω is given as follows:

(12) $$\omega =\omega_{0}-\frac{\omega_{0}-\omega_{e}}{\eta_{\max}}\eta$$

where $\omega_{0}$ is the initial value of inertia weight, $\omega_{e}$ is the final value of inertia weight, $\eta_{\max}$ is the maximum number of iterations, η is the current number of iterations. From Equation (12), by the linear function decreasing inertia weight, it is easy to obtain better global search ability and make particles enter the area around the optimal value early in the iteration process, and it is easy to obtain better local search ability and the solutions close to the optimum value late in the iteration process. Since the value of the inertia weight is mainly determined based on the iteration number, this strategy is relatively simple and has fast convergence compared to other methods.

### 4.3. Crossover Operator of Genetic Algorithm

In order to expand the search space to obtain the optimum solution and enhance the diversity of particles, the idea of genetic algorithm is integrated into the PSO to avoid the premature convergence and local minimum value. Crossover operator of genetic algorithm is introduced. The crossover operators of the particle’s velocity and position are given as follows:

(13) $$p_{child}^{1}({x^{t}}_{pn})=p_{c}\times p_{parent}^{1}({x^{t}}_{pn})+(1-p_{c})\times p_{parent}^{2}({x^{t}}_{pn})$$

(14) $$p_{child}^{2}({x^{t}}_{pn})=p_{c}\times p_{parent}^{2}({x^{t}}_{pn})+(1-p_{c})\times p_{parent}^{1}({x^{t}}_{pn})$$

(15) $$p_{child}^{1}({v^{t}}_{pn})=\frac{p_{parent}^{1}({v^{t}}_{pn})+p_{parent}^{2}({v^{t}}_{pn})}{\left|p_{parent}^{1}({v^{t}}_{pn})+p_{parent}^{2}({v^{t}}_{pn})\right|}\left|p_{parent}^{1}({v^{t}}_{pn})\right|$$

(16) $$p_{child}^{2}({v^{t}}_{pn})=\frac{p_{parent}^{1}({v^{t}}_{pn})+p_{parent}^{2}({v^{t}}_{pn})}{\left|p_{parent}^{1}({v^{t}}_{pn})+p_{parent}^{2}({v^{t}}_{pn})\right|}\left|p_{parent}^{2}({v^{t}}_{pn})\right|$$

where $p_{child}$ represents the offspring of the particle, $p_{parent}$ represents the parent of the particle, $p_{c}$ represents the crossover probability. $p_{c}$($p_{c}\in [0,1]$) is a random number. Based on the trial and error, 0.2 was found to be a suitable value for $p_{c}$. The two parents are combined to produce two new offspring. If the fitness of offspring is more than the fitness of parents, the offspring will be selected to replace the parents; otherwise, the offspring are discarded.

### 4.4. The Proposed Algorithm Flow

**Figure 1:**
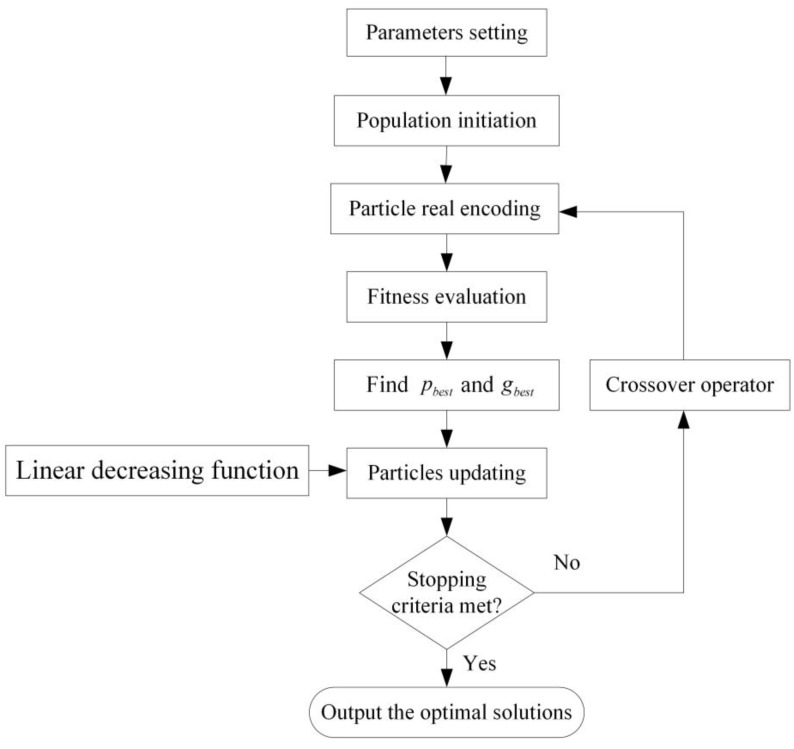
The flow of the proposed algorithm.

The flow of the proposed algorithm is shown in Figure 1. In this flow, the parameters are set in step (1), and the particles are initialized in step (2). Its position is decoded in step (3), its corresponding fitness value is evaluated in step (4), its cognitive and social information is updated in step (5), and then moved by step (6). Step (7) is the controlling step for repeating or stopping the iteration. Step (8) generates a new set of the population. Note that the improvements of this flow from the original PSO take place in step (3), which uses the real encoding method to decode the route in addition to step (6), which introduces a linear decreasing function of the number of iterations, and step (8), which is combined with the crossover operator of genetic algorithm. The proposed algorithm steps are given as follows:

(1) Parameters setting. Define the parameters: acceleration coefficients $k_{1}$ and $k_{2}$, the maximum number of iterations $\eta_{\max}$, the initial value of inertia weight $\omega_{0}$, the final value of inertia weight $\omega_{e}$, a random number $p_{c}$, the number of the particles P, the maximum velocities of the particles $v_{\max}$.
(2) Population initiation. Initialize P particles as a population, generate the *p*th particle with random position ${x^{0}}_{pn}$, velocity ${v^{0}}_{pn}$, and personal best $p_{best_{pn}}^{0}={x^{0}}_{pn}$. Set iteration η=0.
(3) Particle encoding. According to the particle encoding rules, for i=1,2,⋯,p, decode ${x^{t}}_{pn}$ to a set of route ${R^{t}}_{pn}$.
(4) Fitness evaluation. According to Equation (1), compute z, and then evaluate $f_{fitness}$ through Equation (9).
(5) $p_{best}$ and $g_{best}$ updating. Compute $p_{best_{pn}}^{t}$ and $g_{best_{pn}}^{t}$. If $p_{best_{pn}}^{t}<p_{best}$, update $p_{best}=p_{best_{pn}}^{t}$. If $g_{best_{pn}}^{t}<g_{best}$, update $g_{best}=g_{best_{pn}}^{t}$.
(6) Particles updating. Update the velocity and the position of each *p*th particle according to Equation (10).
(7) Termination judgment. If the stopping criterion is met, go to step (9). Otherwise, η=η+1 and go to step (8). The stopping criterion is that $\eta \geq \eta_{\max}$ or finding a better solution. A better solution means that the hierarchical cost objective value is better than that of the best solution found so far.
(8) Crossover operator. Generate random number $p_{c}$. According to Equations (13)–(16), generate a new set of population. Then return to step (3).
(9) Outputting the optimal solution. Decode $g_{best}$ as the best set of vehicle route R and output the optimal solution R.

## 5. Experimental Results

In order to evaluate the performance of the proposed algorithm, we implemented the algorithm in Visual C# under Microsoft Windows-XP on a PC with Intel P4 4 GHz CPU and 2 GB RAM. The VRPTW benchmark problems of Solomon, which have been the most commonly chosen to evaluate and compare all algorithms, are tested in this paper.

### 5.1. Solomon Benchmark Problems

It is well known that Solomon Benchmark problems are the widely used standard test set for VRPTW. Solomon Benchmark derives from the website [64]. The Solomon test set consists of 56 problem instances for each dimension category problem, *i.e.*, 25, 50 and 100 customers. Each of these instances comprises 100 customers. The location of the depot and the customers are given as integer values from the range 0,1,⋯,100 in a Cartesian coordinate system. The distance between two customers is the simple Euclidean distance. It is assumed that the travel times are equal to the corresponding Euclidean distances between the customer locations. One unit of time is necessary to run one unit of distance by any vehicle. Different capacity constraints are considered for the vehicle in each class of instance, as well as the demands from the customers.

The test problems are grouped into six problem types: R1, R2, RC1, RC2, C1, and C2, each containing 8 to 12 instances. In R1 and R2, the customer locations are generated randomly in a given area according to a uniform distribution. C1 and C2 have customers located in clusters. RC1 and RC2 contain a mix of randomly distributed and clustered customers. R1, C1 and RC1 have narrow time windows and the vehicles have only small capacities. Therefore, each vehicle serves only a few customers. In contrast, R2, C2 and RC2- have wider windows and the vehicles have higher capacities. Each vehicle supplies more customers and therefore, compared to the type 1 problems, fewer vehicles are needed. Because the Solomon’s test set represents relatively well different kinds of scenarios, it has often been chosen to evaluate many solution proposals in the literature.

### 5.2. Evaluation of the Results

In order to compare the solutions, four evaluation indexes are presented: the total traveled distance, the average total traveled distance, the CPU runtime and the quality of the solutions. The total traveled distance and the average total traveled distance are the main criteria in the VRPTW and determines the algorithm merits. The CPU runtime describes the algorithm efficiency. The quality of the solutions is the comprehensive index, which denotes the percentage of deviation from the best-known solution. The formula for calculating the percentage of deviation is as follows:

(17) $$z_{dev}=\frac{z-z_{best}}{z_{best}}\times 100\%$$

where $z_{dev}$ is the percentage of deviation from the best-known solution, z is the cost of the current solution, $z_{best}$ is the cost of the best-known solution.

### 5.3. Analysis of the Results

Each of 56 Solomon Benchmark problems is performed by some of the best-reported methods for VRPTW, namely, the genetic algorithm, ant colony optimization [29], PSO [65] and the proposed algorithm. Each problem involves 100 customers, randomly distributed over a geographical area. For each problem, 10 replications of the algorithm are attempted. According to Equation (1), we have adopted a formulation in which the total traveled distance is the optimized objective. Results displayed in the following tables contain the total traveled distance and the number of vehicles used in order to facilitate the comparison of our results with those obtained by hierarchical approaches in which the total traveled distance is the primary objective and, for the same total traveled distance, the secondary objective is number of vehicles, and also with multi-objective formulations that simultaneously consider both objectives [66]. It compares the total traveled distance (TD), the average total traveled distance, the CPU runtime and the quality of the solutions for each of the problems. Both best and average results are presented.

#### 5.3.1. The Total Traveled Distance

The results of TD and the number of vehicles (NV) are shown in Table 2. The published best-known solutions are not obtained by one or a particular class of methods [15,67,68,69,70,71]. Results emphasized in bold in Table 2 represent the new best solutions reached by the proposed algorithm: 1 out of 56 (1.79%). Results emphasized in bold and italic in Table 2 represent the previously best-known solutions that cannot be reached by this algorithm. The results show that many previously best-known solutions from the proposed algorithm have been reached: 31 out of 56 (55.4%). Globally, the TD average results are close to the optimal solutions known in the proposed algorithm. This comparison shows that the results from the proposed algorithm are competitive with other published results. In the genetic algorithm, 27 out of 56 (48.2%) have reached the previously best-known solutions. In PSO, 28 out of 56 (50%) have reached the previously best-known solutions. In ACO, 26 out of 56 (46.4%) have reached the previously best-known solutions. It can be seen that the proposed algorithm is better than the genetic algorithms, ACO and PSO. The efficiency is improved and the results are close to the best-known solutions. It is possible to see that the proposed algorithm continues to be very competitive in terms of total TD.

**Table 2:** The results of the total traveled distance for Solomon’s 100 customers set Problems.

No.	Problem	Best-Known Solution		Genetic				PSO				ACO				The Proposed Algorithm			
				Best		Average		Best		Average		Best		Average		Best		Average	
		TD	NV	TD	NV	TD	NV	TD	NV	TD	NV	TD	NV	TD	NV	TD	NV	TD	NV
**1**	**C101**	828.94	10	828.94	10	856.26	10.20	828.94	10	842.60	10.10	828.94	10	842.60	10.10	828.94	10	842.60	10.10
**2**	**C102**	828.94	10	828.94	10	828.94	10.00	828.94	10	828.94	10.00	828.94	10	857.82	10.10	828.94	10	828.94	10.00
**3**	**C103**	828.06	10	828.06	10	859.88	10.00	828.06	10	828.06	10.00	828.06	10	828.06	10.00	828.06	10	828.06	10.00
**4**	**C104**	824.78	10	824.78	10	824.78	10.00	824.78	10	824.78	10.00	824.78	10	849.79	10.10	824.78	10	824.78	10.00
**5**	**C105**	828.94	10	828.94	10	854.25	10.20	828.94	10	866.91	10.30	828.94	10	904.88	10.60	828.94	10	841.60	10.10
**6**	**C106**	828.94	10	828.94	10	851.78	10.10	828.94	10	897.46	10.30	828.94	10	943.14	10.50	828.94	10	874.62	10.20
**7**	**C107**	828.94	10	828.94	10	856.47	10.20	828.94	10	842.70	10.10	828.94	10	870.23	10.30	828.94	10	842.70	10.10
**8**	**C108**	828.94	10	828.94	10	865.99	10.00	828.94	10	841.29	10.00	828.94	10	853.64	10.00	828.94	10	841.29	10.00
**9**	**C109**	828.94	10	828.94	10	910.27	10.30	828.94	10	856.05	10.10	828.94	10	883.16	10.20	828.94	10	828.94	10.00
**10**	**C201**	591.56	3	591.56	3	602.53	3.30	591.56	3	606.18	3.40	591.56	3	609.84	3.50	591.56	3	598.87	3.20
**11**	**C202**	591.56	3	591.56	3	656.01	3.20	591.56	3	623.78	3.10	591.56	3	623.78	3.10	591.56	3	591.56	3.00
**12**	**C203**	591.17	3	591.17	3	606.19	3.00	591.17	3	604.69	3.00	591.17	3	618.20	3.00	591.17	3	604.69	3.00
**13**	**C204**	590.60	3	590.60	3	704.28	3.30	590.60	3	666.39	3.20	590.60	3	742.17	3.40	590.60	3	628.49	3.10
**14**	**C205**	588.88	3	588.88	3	619.27	3.30	588.88	3	599.01	3.10	588.88	3	609.14	3.20	588.88	3	599.01	3.10
**15**	**C206**	588.49	3	588.49	3	620.28	3.20	588.49	3	604.38	3.10	588.49	3	636.17	3.30	588.49	3	588.49	3.00
**16**	**C207**	588.29	3	588.29	3	610.49	3.00	588.29	3	632.69	3.00	588.29	3	621.59	3.00	588.29	3	599.39	3.00
**17**	**C208**	588.32	3	588.32	3	622.73	3.00	588.32	3	599.79	3.00	588.32	3	611.26	3.00	588.32	3	599.79	3.00
**18**	**R101**	1483.57	16	***1642.87***	20	1645.83	19.50	***1642.87***	20	1645.33	19.50	***1645.79***	19	1647.29	19.00	***1642.87***	20	1645.83	19.50
**19**	**R102**	1355.93	14	***1482.74***	18	1483.75	17.70	***1472.62***	18	1477.95	17.80	***1480.73***	18	1482.21	17.80	***1472.62***	18	1477.14	17.80
**20**	**R103**	1133.35	12	***1292.85***	15	1248.88	14.40	***1213.62***	14	1239.30	14.40	***1213.62***	14	1247.22	14.30	***1213.62***	14	1239.01	13.80
**21**	**R104**	968.28	10	***982.01***	10	995.05	9.60	***1007.24***	9	1007.30	9.00	***982.01***	10	1000.11	9.40	***982.01***	10	992.52	9.70
**22**	**R105**	1262.53	12	***1360.78***	15	1366.87	14.80	***1360.78***	15	1374.14	14.70	***1360.78***	15	1369.99	15.30	***1360.78***	15	1366.42	15.00
**23**	**R106**	1201.78	12	***1249.40***	13	1251.48	12.20	***1241.52***	13	1249.39	12.40	***1251.98***	12	1252.00	12.00	***1241.52***	13	1247.29	12.60
**24**	**R107**	1051.92	11	***1076.13***	11	1094.18	10.70	***1076.13***	11	1094.19	10.50	***1076.13***	11	1096.08	10.70	***1076.13***	11	1088.49	10.70
**25**	**R108**	948.57	10	***963.99***	9	965.42	9.90	***963.99***	9	965.10	9.70	***948.57***	10	960.92	9.60	948.57	10	955.82	9.60
**26**	**R109**	1110.40	12	***1151.84***	13	1181.86	11.60	***1151.84***	13	1186.15	11.40	***1151.84***	13	1190.44	11.20	***1151.84***	13	1164.71	12.40
**27**	**R110**	1080.36	11	1080.36	11	1112.79	10.30	1080.36	11	1105.14	10.50	***1080.36***	11	1107.72	10.50	1080.36	11	1098.14	10.70
**28**	**R111**	987.80	10	***1053.50***	12	1086.43	10.80	***1053.50***	12	1083.13	11.60	***1088.48***	12	1095.07	10.40	***1053.50***	12	1069.14	11.60
**29**	**R112**	953.63	10	***982.14***	9	995.33	10.80	953.63	10	965.03	10.00	953.63	10	987.40	10.60	***960.68***	10	968.58	9.90
**30**	**R201**	1148.48	9	1148.48	9	1208.94	7.70	***1179.79***	9	1231.20	5.50	***1148.48***	9	1212.51	7.30	1148.48	9	1178.39	8.40
**31**	**R202**	1049.74	7	1049.74	7	1086.62	5.70	1049.74	7	1100.68	5.00	***1079.36***	6	1142.69	4.10	1049.74	7	1081.57	5.70
**32**	**R203**	900.08	5	900.08	5	930.23	4.60	***932.76***	7	939.72	3.80	***939.54***	3	941.70	3.00	900.08	5	922.71	4.60
**33**	**R204**	772.33	4	***807.38***	4	828.06	2.40	772.33	4	817.42	2.80	772.33	4	823.17	2.80	772.33	4	801.47	3.40
**34**	**R205**	959.74	4	***970.89***	6	982.66	4.50	***970.89***	6	980.30	4.80	***970.89***	6	989.71	3.60	***970.89***	6	977.95	5.10
**35**	**R206**	898.91	5	898.91	5	906.06	3.40	***906.14***	3	910.24	3.00	***906.14***	3	912.29	3.00	898.91	5	903.89	4.00
**36**	**R207**	814.78	3	814.78	3	844.73	3.40	814.78	3	868.25	2.60	814.78	3	876.51	2.60	814.78	3	836.67	3.00
**37**	**R208**	715.37	3	***725.75***	2	726.61	2.00	***725.42***	4	725.77	3.20	***725.75***	2	726.71	2.00	***723.61***	3	725.50	2.50
**38**	**R209**	879.53	6	879.53	6	892.08	5.40	879.53	6	891.00	5.40	879.53	6	903.21	4.20	879.53	6	891.82	5.10
**39**	**R210**	932.89	7	***954.12***	3	955.06	6.60	***954.12***	3	954.64	5.00	***939.34***	3	952.94	4.78	932.89	7	937.89	6.20
**40**	**R211**	761.10	4	***885.71***	2	892.31	2.30	***885.71***	2	889.53	4.40	***888.73***	5	867.07	2.90	***808.56***	4	824.99	3.90
**41**	**RC101**	1481.27	13	***1660.10***	16	1689.57	14.40	***1623.58***	15	1658.18	15.10	***1639.97***	16	1687.56	14.40	***1623.58***	15	1645.18	15.10
**42**	**RC102**	1395.25	13	***1466.84***	14	1493.53	13.50	***1482.91***	14	1497.28	13.60	***1477.54***	13	1539.85	12.30	***1466.84***	14	1487.10	13.50
**43**	**RC103**	1221.53	10	***1261.67***	11	1263.56	11.00	***1262.02***	11	1262.29	11.00	***1262.02***	11	1264.17	11.00	***1261.67***	11	1262.04	11.00
**44**	**RC104**	1135.48	10	1135.48	10	1135.50	10.00	1135.48	10	1135.50	10.00	1135.48	10	1135.51	10.00	1135.48	10	1135.49	10.00
**45**	**RC105**	1354.20	12	***1518.60***	16	1601.17	15.40	***1518.60***	16	1593.35	14.80	***1629.44***	13	1633.29	13.00	***1618.55***	16	1621.16	15.40
**46**	**RC106**	1226.62	11	***1377.35***	13	1396.59	11.80	***1384.92***	12	1417.25	11.20	***1377.35***	13	1416.49	11.30	***1377.35***	13	1392.80	12.30
**47**	**RC107**	1150.99	10	***1230.48***	11	1254.98	12.60	***1212.83***	12	1240.50	12.30	***1230.48***	11	1258.04	12.80	***1212.83***	12	1226.01	12.00
**48**	**RC108**	1076.81	10	***1117.53***	11	1135.32	10.30	***1117.53***	11	1127.41	10.90	***1117.53***	11	1128.55	10.80	***1117.53***	11	1126.40	10.70
**49**	**RC201**	1134.91	6	***1406.91***	4	1391.43	7.20	***1406.91***	4	1406.91	4.00	***1286.83***	9	1397.23	6.00	***1387.55***	8	1391.43	7.20
**50**	**RC202**	1113.53	8	***1365.57***	4	1250.18	3.60	1113.53	8	1162.75	7.60	1113.53	8	1204.86	6.30	***1148.84***	9	1173.34	7.90
**51**	**RC203**	945.96	5	945.96	5	1034.30	3.40	945.96	5	1032.14	3.40	***1049.62***	3	1052.87	3.00	945.96	5	990.67	4.20
**52**	**RC204**	796.11	4	***798.41***	3	798.69	3.20	***798.41***	3	799.18	3.60	***798.46***	3	799.43	3.80	***798.41***	3	798.67	3.20
**53**	**RC205**	1168.22	8	***1270.69***	7	1276.08	6.40	1168.22	8	1282.01	4.70	1168.22	8	1263.14	6.80	**1161.81**	**7**	1187.56	6.90
**54**	**RC206**	1059.89	7	1059.89	7	1105.75	5.80	***1084.30***	8	1139.24	4.00	1059.89	7	1135.44	4.00	***1059.89***	7	1092.22	6.00
**55**	**RC207**	976.40	7	***999.26***	6	1060.37	3.60	976.40	7	1011.75	5.70	***1053.58***	6	1064.28	3.90	976.40	7	995.65	6.40
**56**	**RC208**	785.93	4	***816.10***	5	824.93	3.60	***816.10***	5	822.41	4.00	***806.87***	5	819.64	4.00	***795.39***	**5**	807.27	4.78

#### 5.3.2. The Average Total Traveled Distance

The results of the average total traveled distance are shown in Table 3. Each entry refers to the best performance obtained with a specific technique over a particular data set. Rows C1, C2, R1, R2, RC1 and RC2 present the average number of vehicles and average total distance with respect to the six problem groups. The last row refers to the cumulative number of routes and traveled distance over all problem instances. The first column describes the various data sets and corresponding measures of performance defined by the average number of routes (or vehicles), and the total traveled distance. The following columns refer to particular problem-solving methods. The performance of the proposed algorithm is depicted in the last column. The results indicate that the proposed algorithm can match the best-known solutions, and it performs better than other algorithms. In addition, the proposed algorithm is the only method that found the minimum number of tours and the comparable traveled distance consistently for all problem data sets.

**Table 3:** The results of the average total traveled distance.

Problem		Best-Known Solution	Genetic		PSO		ACO		The Proposed Algorithm	
			Best	Average	Best	Average	Best	Average	Best	Average
**C1-type**	**NV**	10.00	10.00	10.11	10.00	10.10	10.00	10.21	10.00	10.06
	**TD**	828.38	828.38	856.51	828.38	847.64	828.38	870.37	828.38	839.28
**C2-type**	**NV**	3.00	3.00	3.16	3.00	3.11	3.00	3.19	3.00	3.05
	**TD**	589.86	589.86	630.22	589.86	617.11	589.86	634.02	589.86	601.29
**R1-type**	**NV**	11.67	13.00	12.69	12.92	12.63	12.92	12.57	13.08	12.78
	**TD**	1128.18	1193.22	1202.32	1184.84	1199.35	1186.16	1203.04	1182.04	1192.76
**R2-type**	**NV**	5.18	4.73	4.36	4.91	4.14	4.55	3.66	5.36	4.72
	**TD**	893.90	912.31	932.12	915.56	937.16	914.99	940.77	899.98	916.62
**RC1-type**	**NV**	11.13	12.75	12.38	12.63	12.36	12.25	11.95	12.75	12.50
	**TD**	1255.27	1346.01	1371.28	1342.23	1366.47	1358.73	1382.93	1351.73	1362.02
**RC2-type**	**NV**	6.13	5.13	4.60	6.00	4.63	6.13	4.73	6.38	5.82
	**TD**	997.62	1082.85	1092.72	1038.73	1082.05	1042.13	1092.11	1034.28	1054.60
**All**	**NV**	449	465	452.40	472	448.70	466	441.88	483	466.68
	**TD**	53,568.46	55,959.11	57,143.53	55,511.30	56,854.75	55,679.89	57,490.75	55,346.67	56,092.75

#### 5.3.3. The Average Central Processing Unit (CPU) Runtime

The results of the average CPU runtime are listed in Table 4. Rows C1, C2, R1, R2, RC1 and RC2- present the average CPU runtime with respect to the six problem groups. From Table 4, for the same instance, ACO is almost the most time consuming; the genetic algorithm takes second place; the PSO takes third place; and the proposed algorithm is last. The result shows that the proposed algorithm is highly efficient and suitable to be used in real-life problems to generate good solutions for further improvement. The average CPU runtime required to solve each type of problem set is between 62 and 149 milliseconds by the proposed algorithm, which is much faster than other algorithms. It can be seen that the proposed algorithm is very effective. This happened because crossover in GA is done between random chromosomes, whereas in the proposed algorithm, crossover is done between a particle’s chromosome and best chromosome. Therefore, the proposed algorithm does not only give a better result, but it also reaches convergence results faster than other methods.

**Table 4:** The results of the average CPU runtime.

Problem	Genetic	PSO	ACO	The Proposed Algorithm
C1e	98	85	111	62
C2	312	231	338	135
R1	112	81	124	60
R2	309	273	553	182
RC1	79	80	82	58
RC2	297	257	333	149

#### 5.3.4. The Quality of the Solutions

According to Equation (17), the quality of the solutions is shown in Table 5. Here z is the average total traveled distance. Rows C1, C2, R1, R2, RC1 and RC2 present the average $z_{dev}$ with respect to the six problem groups. The last row refers to the cumulative $z_{dev}$ over all problem instances. It can be seen that, with the proposed method, the quality of the results is between 1.32% and 8.17% with average quality equal to 4.07%; in the ACO, the quality of the solutions is between 5.07% and 9.75% with average quality equal to 6.95%; in PSO, the quality of the solutions is between 2.32% and 8.53% with average quality equal to 5.63%; in the genetic algorithm, the quality of the solutions is between 3.39% and 8.96% with average quality equal to 6.29%. The improvement in the quality of the solutions was achieved with the addition of the crossover operator of genetic algorithm. The reason is that, now, the particles moved in a more fast and efficient way to their local optimum or to the global optimum solution (to the best particle in the swarm). It is proved that the addition of the evolution of the population phase before the individuals used in the next generation improves the results of the algorithm, especially in the large-scale vehicle routing instances which are more difficult and time consuming.

**Table 5:** The result of $z_{dev}$.

Problem	Genetic	PSO	ACO	The Proposed Algorithm
C1-type	3.39%	2.32%	5.07%	1.32%
C2-type	6.84%	4.62%	7.48%	1.94%
R1-type	6.28%	5.98%	6.33%	5.36%
R2-type	4.44%	4.93%	5.29%	2.62%
RC1-type	8.89%	8.53%	9.75%	8.17%
RC2-type	8.96%	7.92%	8.95%	5.30%
All	6.29%	5.63%	6.95%	4.07%

From the results shown in Table 2, Table 3, Table 4 and Table 5, it is observed that the proposed algorithm is fast and produces good solutions, which are only slightly less accurate than the best-known results. This comparison also shows that the results from the proposed algorithm are competitive with other published results. The experimental results reveal that the proposed algorithm is highly capable at minimizing the total travel distance. In particular, it is obvious that the proposed algorithm has great advantages for the solved problem size over the genetic algorithm, PSO and ACO. The reason is that, now, the particles moved in a more fast and efficient way to their local optimum or to the global optimum solution (to the best particle in the swarm). After each environment change (peak movement), the particles are placed on a point far from the optimum through the inertia weight function. The particles take few iterations to reach this point. At the same time, the particle’s coding can be ensured that a particle is always able to generate a new feasible solution. 

## 6. Conclusions

This paper proposes a genetic and PSO algorithm for VRPTW. Moreover, this algorithm was applied to the Solomon Benchmark problems and produced satisfactory results. Compared to other approaches, experimental results indicate that the proposed algorithm is fast and possesses a relatively accurate results. The proposed algorithm has proved to be an effective and competitive algorithm for the optimization problems due to its easy implementation, inexpensive computation and low memory requirements. Three major contributions are as follows:

(1) The real encoding method avoids the complex encoding and decoding computation burden.
(2) A linear decreasing function of the number of iterations in PSO have a flexible and well-balanced mechanism to enhance and adapt to the global and local exploration abilities, which can help find the optimal solution with the least number of iterations.
(3) The crossover operator of the genetic algorithm is introduced to generate a new population guaranteeing that the offspring inherits good qualities from this parent. The crossover operator avoids premature convergence and local minimum value and increases the diversity of particles.

Although the combination of the genetic algorithm and PSO for VRPTW obtains satisfactory achievements, there is still some room for improvement, such as how to effectively construct the initial solution and how to precisely judge the local optimal solution. Further research applying the proposed algorithm to other VRPTW variants will be carried out in our future work.
